# Supplementary material for: Helicobacter pylori Stress-Response: Definition of the HrcA Regulon
Source: Microorganisms. 2019 Oct 11;7(10):436. doi: 10.3390/microorganisms7100436 (PMC6843607; doi:10.3390/microorganisms7100436)
Supplement: Supplementary file 1 [file microorganisms-07-00436-s001.zip › Supplementary Table S1.docx]

| **Oligonucleotide name** | **Sequence (5’ – 3’)** |
| --- | --- |
| 16S RT F | GGAGTACGGTCGCAAGATTAAA |
| 16S RT R | CTAGCGGATTCTCTCAATGTCAA |
| HPG27_RS00070_F | TCACGCCATCTTTGGTGATGCTTG |
| HPG27_RS00070_R | TGAAGGCGTGAGACAACTCCATGA |
| HPG27_RS01500_F | ATTAGCGTTGGCGTGTTG |
| HPG27_RS01500_R | GCATAGAAATCCCGGCTAAAG |
| HPG27_RS03015 _F | AATCATAGAGCCGGTGGATATG |
| HPG27_RS03015 _R | GAAACAGGGCAAGGCAAATAG |
| HPG27_RS03495 _F | TGGTCCTGTAACCGACTATG |
| HPG27_RS03495 _R | TCAGATAGGGTGTGGTTTCTC |
| HPG27_RS00595_F | TGAAGACGGATTAAGGGCTAAG |
| HPG27_RS00595_R | CGCTTCTATCACGCAATCAATC |
| HPG27_RS00600_F | AAACTTGACTGAAGTGGGATTG |
| HPG27_RS00600_R | GCCAGCGCTTGTAGAAATAC |
| HPG27_RS01480_F | GCGTCTAAACCCAATGAAGTC |
| HPG27_RS01480_R | CGCTGATTTGTTGGTAGATGG |
| HPG27_RS01870_F | AGCCTTTCAAACCCTAAAGC |
| HPG27_RS01870_R | CCCTCAAATCATCGCTCAAC |
| HPG27_RS02255_F | ATGGAATTTAGGCGGAAGAATG |
| HPG27_RS02255_R | CCTACATCTTTGCTGCCTTG |
| HPG27_RS02740_F | ATGACGATCACGACTCTATCC |
| HPG27_RS02740_R | ACAAGCCTCCAGCAAATAAC |
| HPG27_RS03665_F | CAAACCACCACCCAAGAAAG |
| HPG27_RS03665_R | GTGTTGCCCTCTTTGGTTAAG |
| HPG27_RS04430_F | AATCCCGCTAAAGATCAACAAG |
| HPG27_RS04430_R | GGTTTCATGCTCATGGTTCTC |
| HPG27_RS05560_F | GAGAGTCCTTGCTTGCTTTG |
| HPG27_RS05560_R | GGGATGATTTGTTGGATGAGC |
| HPG27_RS05740_F | GAGGGAATACAGCTTTGTGATAC |
| HPG27_RS05740_R | CCACGCAATACACCTCAAC |
| HPG27_RS06935_F | CCGTTTATGACAGCGAGTTTATC |
| HPG27_RS06935_R | TTGCTCGCATAGTAGCATTTG |

**Table S1 -** List of oligonucleotides used in this study.
